# Treating vitamin D deficiency in children with type I diabetes could improve their glycaemic control

**DOI:** 10.1186/s13104-017-2794-3

**Published:** 2017-09-07

**Authors:** Dinesh Giri, Dona Pintus, Girvan Burnside, Atrayee Ghatak, Fulya Mehta, Princy Paul, Senthil Senniappan

**Affiliations:** 1 Department of Paediatric Endocrinology & Diabetes, Alder Hey Children’s NHS Foundation Trust, Liverpool, UK; 2 Institute of Child Health, University of Liverpool, Liverpool, UK; 3 Department of Biostatistics, University of Liverpool, Liverpool, UK

**Keywords:** Vitamin D deficiency, HbA1C, Type 1 diabetes mellitus

## Abstract

**Background and aims:**

The relationship between vitamin D deficiency and type I DM is an ongoing area of interest. The study aims to identify the prevalence of vitamin D deficiency in children and adolescents with T1DM and to assess the impact of treatment of vitamin D deficiency on their glycaemic control.

**Methods:**

Retrospective data was collected from 271 children and adolescents with T1DM. The vitamin D deficient (25(OH)D <30 nmol/L) and insufficient (25(OH)D 30–50 nmol/L) patients were treated with 6000 units of cholecalciferol and 400 units of cholecalciferol, once daily for 3 months respectively. HbA1c and 25(OH)D concentrations were measured before and at the end of the vitamin D treatment.

**Results:**

14.8% from the whole cohort (n = 271) were vitamin D deficient and 31% were insufficient. Among the children included in the final analysis (n = 73), the mean age and plasma 25(OH)D concentration (±SD) were 7.7 years (±4.4) and 32.2 nmol/l (±8.2) respectively. The mean 25(OH)D concentration post-treatment was 65.3 nmol/l (±9.3). The mean HbA1c (±SD) before and after cholecalciferol was 73.5 mmol/mol (±14.9) and 65 mmol/mol (±11.2) respectively (p < 0.001). Children with higher pre-treatment HbA1c had greater reduction in HbA1c (p < 0.001) and those with lower 25(OH)D concentration showed higher reduction in HbA1c (p = 0.004) after treatment.

**Conclusions:**

Low 25(OH)D concentrations are fairly prevalent in children and adolescents with T1DM, treatment of which, can potentially improve the glycaemic control.

## Background

The understanding of the effects and role of vitamin D and its analogues in the functioning of body tissues, systems and organs has improved substantially over the last decade. Vitamin D deficiency is generally accepted as, circulating 25-hydroxyvitamin D concentration <30 nmol/L [1]. Vitamin D functions in the body through both by an endocrine mechanism, which is to regulate the absorption of calcium and also by an autocrine mechanism by facilitating the gene expression [2]. Vitamin D deficiency has been implicated in a variety of chronic diseases, including bone mineral disease, autoimmunity, cancer, and diabetes [3].

The potential extra-skeletal role of vitamin D has been a rich area of interest and research over the last decade. Some studies have associated vitamin D deficiency with β-cell dysfunction and insulin resistance with a consequent development of type 2 diabetes in the adult population [4–6]. In children with type I diabetes mellitus (T1DM), the prevalence of vitamin D deficiency has been shown to be high when compared to the general population [7–9]. However, another study found that although vitamin D deficiency/insufficiency is present in a substantial proportion of youth with both T1DM and T2DM diabetes, particularly minorities, the prevalence is similar to that in youth without diabetes [10].

Furthermore, a Finnish study reported that there was a fourfold increased risk of development of T1DM in children who had rickets [11]. Children who received 2000 IU of vitamin D per day from 1 year of age and followed through adulthood had an 80% reduced risk of developing T1DM [11]. An improvement in the underlying T cell regulatory defect and glycaemic status was demonstrated in patients with TIDM and vitamin D deficiency with vitamin D treatment [12]. Data from these studies suggest that there is a possible correlation between vitamin D deficiency and the risk of both T1DM and type 2 diabetes mellitus. However, there is a paucity of studies examining the direct effects of vitamin D treatment in children with T1DM and vitamin D deficiency.

In this study, we aimed to study the prevalence of vitamin D deficiency in T1DM and the effect of vitamin D treatment on glycaemic control (HbA1C) in children and adolescents with T1DM and vitamin D deficiency.

## Methods

This a retrospective study, whereby, we looked into the electronic medical records and collected data from 271 children and young people with T1DM, attending the paediatric outpatient diabetes clinic at a tertiary children’s hospital. The vitamin D status (D2 + D3) was assessed as a part of their annual review. Vitamin D status was classified according to the Global Consensus Recommendations on Prevention and management of Nutritional Rickets [1]. A plasma 25(OH)D concentration of <30 nmol/l was considered as deficiency, a concentration between 30 and 50 nmol/l as insufficiency, and a plasma concentration above 50 nmol/l as normal (sufficient) [1]. Patients with insufficient vitamin D concentrations were treated with 400 units of cholecalciferol for 3 months. Patients with vitamin D deficiency were treated with 6000 units of cholecalciferol once a day for 3 months in total as per the hospital guidelines. Patients had their HbA1C measured before and after the completion of treatment with cholecalciferol and plasma 25(OH)D concentration was measured after the completion of treatment with vitamin D. The data collection included co-morbidities associated with T1DM (coeliac disease, thyroid problems etc.) and the type of insulin regimen [multiple daily injections (MDI) and continuous subcutaneous insulin infusion (CSII)].

Serum 25(OH)D level was measured by Tandem Mass Spectrometry method (Thermo TSQ Vantage Tandem Mass Spectrometer). HbA1c levels were measured by monoclonal antibody agglutination method (Siemens DCA Vantage analyser). The statistical analyses were performed with SAS software, version 9.3 (SAS Institute Inc., Cary, NC, USA). Multiple regression analysis was used to study the relationship between 25(OH)D concentration and HbA1c, adjusting for the effects of age and presence of comorbidities. The results were expressed as mean ± SD. A p value of less than 0.05 was considered as statistically significant.

## Results

40 (14.8%) from the cohort of children with T1DM (n = 271) had vitamin D deficiency and 84 (31.0%) of the children were vitamin D insufficient. 147 (54.2%) children had normal serum concentrations of 25(OH)D (>50 nmol/l) and therefore were excluded from the study. Among the vitamin D deficient and insufficient children (n = 124), 73 children had their HbA1C and plasma 25(OH)D concentration measured prior to the commencement and after the completion of treatment with cholecalciferol and were therefore included in the study. Among the included subjects (n = 73), 91% (n = 66) were White British, 5.4% were Somalian origin and the rest were of Asian origin. The patient characteristics are summarised in Table 1. Sixty-two children (84.9%) were on multiple daily injections (MDI) and 11 children (15.1%) were on continuous subcutaneous insulin infusion (CSII) via pump. Nine children (12.3%) had co-morbidities associated with T1DM [coeliac (n = 5) and hypothyroidism (n = 4)].

**Table 1:** Patient characteristics of those included in the study

	n = 73
Age (mean, SD)	(7.7, 4.4)
Ethnicity	White British: 66 (91%) Somalian: 4 (5.4%) Asian: 3 (3.9%)
Vitamin D level (nmol/L) before treatment (mean, SD)	(32.2, 8.2)
Vitamin D category	
Deficient	30 (41.1%)
Insufficient	43 (58.9%)
Vitamin D level (nmol/l), after treatment (mean, SD)	(65.3, 9.3)
HbA1c(mmol/mol) before vitamin D treatment (mean, SD)	(73.5, 14.9)
HbA1c (mmol/mol) after vitamin D treatment (mean, SD)	(65.0, 11.2)
Change in HbA1c (mmol/mol) (mean, SD)	(8.5, 12.5) [p < 0.001]
Type of insulin regimen	
MDI	62 (84.9%)
Pump	11 (15.1%)
Comorbidities	
Yes	9 (12.3%)
No	64 (87.7%)

The mean age (±SD) was 7.7 years (±4.4) and the mean plasma 25(OH)D concentration was 32.2 nmol/l (±8.2). Thirty children (41.1%) from the study group (n = 73) were vitamin D deficient and 43 (58.9%) were vitamin D Insufficient. The mean HbA1C (±SD) prior to commencing treatment and after the completion of treatment in the deficient and the insufficient groups were 73.5 mmol/mol (±14.9) and 65 mmol/mol (±11.2) respectively (p < 0.001). The mean plasma 25(OH)D concentration after the completion of treatment was 65.3 nmol/l (±9.3). Using a multiple regression analysis, we found that children with higher pre-treatment HbA1c had a significantly greater reduction in HbA1C (p < 0.001) after treatment with cholecalciferol. The children with lower vitamin D levels showed a significantly higher reduction in HbA1C (p = 0.004) after treatment with cholecalciferol (Table 2). There was no significant statistically demonstrable relationship between the post treatment HbA1c with age (p = 0.830) and co-morbidities (p = 0.384) (Table 2).

**Table 2:** Multiple regression table showing factors influencing post-treatment HbA1c adjusted for age and co-morbidities

Variable	Regression co-efficient	95% CI	p value
Pre-HbA1c	0.56	(0.46, 0.67)	<0.001
Pre-treatment vitamin D	−0.27	(−0.46, −0.09)	0.004
Comorbidities	−2.11	(−6.88, 2.66)	0.384
Age	−0.04	(−0.39, 0.31)	0.830

## Discussion

In the recent years it has become evident that the pancreatic tissue, especially the β-cells express the vitamin D receptor(VDR) and variations in the genes controlling the vitamin D metabolism and expression of VDR have been implicated in the pathogenesis of diabetes(T1DM and type 2 DM) [13]. Furthermore, animal studies on mice islets have demonstrated an impaired insulin secretion to glucose stimulation in vitro in vitamin D deficient mice that improved with administration of 1,25(OH)2 D3 supplementation [13]. Studies in adults have shown that there is a correlation between the severity of vitamin D deficiency and the frequency of T1DM and supplementing with vitamin D supplementation like cod-liver oil during the first year of life reduces the risk of childhood onset T1DM [11, 14].

A recent meta-analysis examining the correlation of serum vitamin D level with T1DM in children found that the serum vitamin D level was significantly lower than in healthy control population [15]. Our study found that 14.7% of the children with T1DM had vitamin D deficiency, which is similar to the results reported by Svoren et al. who showed that 15% of the children and young people with T1DM had vitamin D deficiency [16]. Zipitis et al. showed in their meta-analysis that vitamin D supplementation in infants significantly reduced the risk of developing T1DM when compared to those who were not supplemented [17]. Data available from retrospective studies show that vitamin D supplementation reduces the risk of T1DM. Majority of these studies show that there is a dose-dependent, positive correlation between regular vitamin D supplementation in early life and a reduced risk for T1DM and development of diabetes-related autoantibodies, whereas vitamin D deficiency in early life is associated with a higher risk of T1DM later in life [18].

There is paucity of data demonstrating the effects of vitamin D supplementation on diabetes control. Clinical trials with low dose 1,25(OH)D3 (0.25 µg) supplementation in T1DM have not shown any significant improvements in insulin requirements in adult subjects [19]. In our study, we had demonstrated that there is a significant improvement in the glycaemic control (measured by HbA1c) on treating the vitamin D deficiency in children and adolescents with T1DM. We have shown that the glycaemic control improves significantly with normalization of vitamin D concentrations. Further, we have shown that the group of children with the poorest glycaemic control and those with the lowest 25(OH)D levels demonstrated a higher improvement in their HbA1C after treatment with cholecalciferol.

Receptors for 1,25(OH)_2_D3 are found in insulin producing β -cells, in target organs of insulin action (liver, skeletal muscle and adipose tissue), as well as in all cells of the immune system. 1,25(OH)_2_D3 has been shown to improve the β-cell function, increase the insulin sensitivity of target tissues and protect the β-cells from immune attacks from inflammatory macrophages, dendritic cells, and a variety of T cells [19]. In our study, an improvement in the vitamin D concentration following treatment of the existing deficiency potentially led to better insulin action thereby improving glycaemic control. The improved concentrations could have also had a potential influence in improving endogenous residual insulin secretion form pancreas.

Our study is limited by the fact that due to its retrospective nature, other factors influencing the glycaemic control such as insulin dosage, compliance, length of diagnosis, BMI (body mass index) of the subjects at various points, psychosocial factors could not be taken into account. The lack of control group is also a shortcoming in our study. The sampling season of 25(OH)D concentration could not be assessed and we acknowledge the fact that plasma concentrations of 25(OH)D may vary depending on the time of the year. Despite these shortcomings, our study highlights the fact that there is a high prevalence of vitamin D deficiency and insufficiency in children and adolescents with T1DM. With the available data from our study, we have also demonstrated that there is a relationship between plasma 25(OH)D concentration with HbA1C noted by the improvement in HbA1C after the treatment of vitamin D deficiency.

## Conclusions

The receptors for 1,25(OH)_2_D3 are present in many cells and tissues including the pancreatic β-cells. It is therefore convincing to note from the literature that there is a potential role of vitamin D in the pathogenesis of DM and a potential role in the prevention of T1DM. Studies have suggested that there is a high prevalence of vitamin D deficiency in children and adolescents with T1DM. Our study, despite its limitations due to its retrospective nature, is in line with the suggestion that there is a potential underlying relationship between vitamin D and glycaemic control in diabetes. Therefore, it will be important to be aware of the fact that vitamin D deficiency is common in diabetes and treatment of the same may help in improving the glycaemic control. Further long term prospective randomized control interventional trials will be useful to understand the long-term effect of vitamin D supplementation on glycaemic control in children and adolescents with TIDM.

## Abbreviations

T1DM: type I diabetes mellitus
25(OH)D: 25-hydroxy vitamin D
1,25(OH)2 D3: 1,25-dihydroxy vitamin D
SD: standard deviation
